# Development of the Experiences of Sex Work Stigma Scale Using Item Response Theory: Implications for Research on the Social Determinants of HIV

**DOI:** 10.1007/s10461-021-03211-1

**Published:** 2021-03-17

**Authors:** Deanna Kerrigan, Tahilin S. Karver, Clare Barrington, Wendy Davis, Yeycy Donastorg, Martha Perez, Hoisex Gomez, Jessie Mbwambo, Samuel Likindikoki, Catherine Shembilu, Andrea Mantsios, S. Wilson Beckham, Noya Galai, Kitty S. Chan

**Affiliations:** 1 Department of Prevention and Community Health, Milken Institute School of Public Health, George Washington University, 950 New Hampshire Avenue NW, Washington, DC, 20052 USA; 2 Department of Health Behavior, University of North Carolina, Chapel Hill, USA; 3 Department of Health, Behavior and Society, Johns Hopkins University, Baltimore, USA; 4 Department of Epidemiology, Johns Hopkins University, Baltimore, USA; 5 Instituto Dermatologico Dominicano y Cirugia de Piel (IDCP), Santo Domingo, Dominican Republic; 6 Muhimbili University of Health and Allied Sciences (MUHAS), Dar es Salaam, Tanzania; 7 Public Health Innovation & Action, New York, NY USA; 8 University of Haifa, Haifa, Israel; 9 Medstar Health Research Institute, Washington DC, USA

**Keywords:** HIV, Sex work, Intersectional, Stigma, Scale, IRT, Reliability, Validity

## Abstract

While HIV stigma has received significant attention, limited work has been conducted on the measurement of intersecting stigmas. We developed the Experiences of Sex Work Stigma (ESWS) scale in the Dominican Republic (DR) and Tanzania. We conducted in-depth interviews with 20 female sex workers (FSW) per country to identify scale domains followed by cognitive debriefing interviews to assess content validity. Items were administered in a survey to FSW in DR (n = 211) and Tanzania (n = 205). Factor analysis established four sex work stigma domains including: shame (internalized), dignity (resisted), silence (anticipated) and treatment (enacted). Reliability across domains ranged from 0.81 to 0.93. Using item response theory (IRT) we created context-specific domain scores accounting for differential item functioning between countries. ESWS domains were associated with internalized HIV stigma, depression, anxiety, sexual partner violence and social cohesion across contexts. The ESWS is the first reliable and valid scale to assess multiple domains of sex work stigma and can be used to examine the effects of this form of intersectional stigma on HIV-related outcomes across settings.

## Introduction

From the beginning of the global HIV pandemic, stigma has been one of the foremost impediments to ensuring access to effective, rights-based HIV prevention and treatment services and, in turn, to reducing the rate of new infections and to decreasing morbidity and mortality among people living with HIV (PLHIV) [1–8]. Forty years into the epidemic, the goal to “end AIDS” [9] is still an aspiration, despite the availability of highly effective biomedical technologies [10]. This challenge remains, in large part, due to our inability to adequately assess and address the role of stigma as a social determinant of health [11], including its impact on the significantly heightened risk and suboptimal HIV care and treatment outcomes observed in key populations across geographic settings [12, 13].

While significant attention has been focused on the assessment of HIV-related stigma [14–16], limited measurement research has been conducted regarding other intersecting stigmas such as those related to sexual orientation, substance use and sex work [14, 15, 17–19]. Sex work stigma has received the least attention [16]. Only a few aggregate measures exist in the peer-reviewed literature to assess occupational stigma among sex workers. These measures have focused on limited aspects of sex work stigma such as perceived sex work stigma or concerns about disclosing one’s occupation to others [20] and internalized sex work stigma or feelings of personal shame associated with sex work [21, 22], potentially neglecting other important dimensions of sex work stigma.

In our own work in the Dominican Republic (DR) and Tanzania, we previously adapted an established HIV stigma scale [23] to measure internalized and enacted sex work stigma [24] and demonstrated their association with HIV outcomes such as retention in care and adherence to ART among cisgender female sex workers (FSW) [25–30]. Neither our adapted measures nor the aforementioned sex work stigma measures from India and the United States were based on formative research allowing sex workers themselves to describe the most salient dimensions and dynamics of their experiences of sex work stigma in the context of their daily lives. As these prior efforts were limited to individual settings, they also left unanswered questions regarding whether and how the latent construct of sex work stigma may be comparable or vary across contexts. An additional gap in the measurement research conducted on sex work stigma has been the limited use of theory to deliberately guide a multi-step scale development process.

The Experiences of Sex Work Stigma (ESWS) scale presented here seeks to address these gaps by generating a reliable and valid scale guided by the voices and experiences of sex workers and critical social theory. In addition, this study aims to empirically test the measurement equivalence of the new scale across two distinct cultural settings. The DR and Tanzania were selected as study sites to explore these specific aims given the ability to integrate these questions into existing cohorts of FSW, as well as longstanding partnerships with sex worker communities in each country. By identifying and accounting for any measurement-related differences, scores will better reflect true differences and allow valid inferences to be made across these settings. In this manuscript we describe the conceptualization, development and performance of a survey instrument, produced using mixed methods and item response theory (IRT), that can assess multiple domains of sex work stigma across contexts.

### Theoretical Orientation

In conceptualizing stigma we drew from Foucault’s work on governmentality, which he defined as the “conduct of conduct,” and he further described as the tension that exists between the “technologies of domination of others” and “technologies of the self” [31]. Acknowledging such tensions, we were interested in exploring activities aimed at affecting or “disciplining” the behavior of sex workers at multiple levels, including within oneself, and between the self and other individuals, social institutions and communities, and the state [32]. As applied to stigma research, the processes mentioned above have allowed for important insights into how “stigmatized” or discredited social identities and groups are constructed within the broader context of actions to reproduce existing power relations (33). Foucault asserted that the maintenance of these structures, which perpetuate stigma and disadvantage, require both self-discipline, through experiences and behaviors such as shame and avoidance, and social discipline, through discriminatory practices and policies that limit socio-economic inclusion [34].

Historically more attention has been placed on Foucault’s work regarding the structural constraints placed on individual agency by the techniques of domination and discipline [35, 36]. Much less attention has focused on his work related to the possibilities of “resistance” in which individuals may create new subjectivities, freeing themselves, to some extent, from certain forms of social control, including stigmatizing identities, and through acts of individual resistance, engage with broader processes of collective action and social change to challenge restrictive norms and inequitable structures [37, 38].

## Methods

### Study Design

The longitudinal observational study, “Stigma, cohesion and HIV outcomes among vulnerable women across epidemic settings” (R01MH110158) is being conducted with FSW living with HIV in the DR and Tanzania during the period 2016–2021. The study integrates biologic, survey, and qualitative data to obtain a holistic understanding of the social determinants of HIV outcomes among FSW living with HIV in these countries. In both settings, women were eligible to participate if they were 18 years or older, had a confirmed HIV-positive diagnosis and reported exchanging sex for money in the last month prior to their enrollment in the study. This analysis focuses on the first aim of the study which was to develop a valid and reliable measure of sex work stigma informed by qualitative and quantitative data collected between 2017 and 2019. In both settings, existing HIV-positive cohorts of FSW were augmented and re-enrolled and consented into the current study. Sampling methods for each cohort are described below.

### Study Sites and Participants

The HIV epidemic in the DR is concentrated among key populations, including FSW [39, 40]. The most recent estimate of national HIV prevalence among FSW was 4.0% [41], which is nearly six times the 0.7% overall national adult prevalence [42]. We established a cohort of FSW living with HIV in the greater Santo Domingo area of the DR in 2011 as part of an implementation science project called *Abriendo Puertas* (opening doors) focused on improving HIV care and treatment outcomes. Initial recruitment took place predominantly by FSW peer navigators, complemented by recruitment of FSW living with HIV by other key informants, such as HIV clinical care providers, and study participants.

In Tanzania, our work has been conducted in the Iringa region of the country, where HIV prevalence is significantly higher (9.1%) than the overall national prevalence (5.0%) [43]. We established a longitudinal cohort of FSW in Iringa in 2015 as part of a randomized trial of a community-driven intervention called Project *Shikamana* (Let’s Stick Together) which sought to reduce HIV incidence and improve care continuum outcomes. In that study, we found a baseline HIV prevalence of 40.9% among venue-based FSW entering the cohort at that time, who were recruited using time location sampling (TLS) [44].

### Data Collection Procedures

All study visits occurred in private offices at the Instituto Dermatologico Dominicano y Cirugía de Piel (IDCP) in Santo Domingo in the DR or the Muhimbili University of Health and Allied Sciences (MUHAS) in the Iringa Region of Tanzania. The study was approved by the Institutional Review Boards of the Johns Hopkins Bloomberg School of Public Health in the United States, IDCP and the Consejo Nacional de Bioética (CONABIOS) in the DR and MUHAS and National Institute of Medical Research (NIMR) in Tanzania. All participants provided informed consent and were compensated ~ USD$ 5 per study visit .

#### In-depth Interviews

In order to understand the nature and dimensions of sex work stigma as experienced by FSW, we first conducted in-depth interviews with 20 women from each of the cohorts described above in the DR and Tanzania. We explored participants’ perceptions of sex work, including how they feel about their work and how others react to their work, their communication regarding sex work, and experiences of and responses to sex work stigma. Interviews were conducted in Spanish or Swahili by a trained interviewer, audiotaped and transcribed. An analytic summary was developed for each interview. We coded interviews for salient sex work stigma domains that emerged based on participants’ lived experiences, and potential scale items per domain using ATLAS.ti©.

Findings from the in-depth interviews identified three initial sex work stigma domains which were salient across both settings, and mirrored similar domains to the HIV Stigma Framework [14], including: internalized (participants’ feelings towards themselves in relation to sex work, including both positive and negative feelings); anticipated (participants’ activities in relation to communicating or disclosing their participation in sex work to others); and enacted (participants descriptions of how they were treated by others for being a sex worker). While the three domains were shared across settings, cross-country variation was documented with regards to relevant items within a domain.

#### Cognitive Debriefing Interviews

Prior to utilizing specific items generated per domain as part of FSW cohort surveys, we sought to assess their understandability and refine possible response options. As such, all potential items associated with the three initial domains identified through in-depth interviews were included in cognitive debriefing interviews with the same 20 FSW in both settings and assessed for content validity. This included a total of 40 items: 10 for internalized, 13 for anticipated and 17 for enacted. We also explored participants’ preferred response options (e.g. 5-, 4- or 3-point Likert scale or dichotomous: yes/no). Cognitive debriefing interviews guided final item selection, refinement of language for framing questions, and appropriate response options for each sex work stigma domain. During this process items were both dropped and added. We noted that positive valence items related to the internalized sex work stigma domain were well-received by participants, particularly in the DR, and as a result two additional positive items were added after cognitive debriefing interviews (e.g. “I feel valued” in relation to sex work, underscoring the importance of income from sex work often sustaining the family).

This iterative process led to a total of 32 items: 12 for internalized, 8 for anticipated and 12 for enacted sex work stigma and the following final questions and response options.

##### Internalized

I would like to know how you feel about sex work. Thinking about the last 6 months, for each phrase, I would like to know if you have you felt like this always, sometimes or never.

##### Anticipated

I would like to know how you speak about your experience with sex work with people around you. Thinking about the last 6 months, for each phrase, I would like to know if it is something that you have done always, sometimes or never.

##### Enacted

Thinking about the last 6 months, have people around you done the following things because of your sex work. Please tell me if it is something that has been done to you always, sometimes or never.

#### Survey Assessments

Once the scale items were finalized on the basis of feedback generated through the cognitive debriefing interviews, they were included as questions within the socio-behavioral survey administered to FSW cohort participants (DR, n = 211; Tanzania, n = 205). The survey included the 32 items related to the three initial sex work stigma scale domains, and the following measures hypothesized to be related to sex work stigma that were used to assess construct validity or how well the scale measured the construct of interest, prior to assessing its predictive validity in terms of HIV outcomes.

##### Depression

We assessed depression using the 9-item Patient Health Questionnaire (PHQ-9) [45], which characterizes the level of depression over the last 2 weeks. The measure was dichotomized as “minimal/no/mild depression” (0–9) vs. “moderate/severe depression” (10–27). The PHQ-9 had an alpha of 0.85 in DR and 0.86 in Tanzania.

##### Anxiety

We assessed anxiety using the Hospital Anxiety and Depression Scale (HADS) [46], which contains 7 items related to anxiety over the last week. The measure was dichotomized as “normal” (0–7) vs. “borderline abnormal/abnormal anxiety level” (8–21). The HADS-A had an alpha of 0.84 in the DR and 0.76 in Tanzania.

##### Internalized HIV Stigma

We utilized the Berger HIV Stigma Scale [23] to assess internalized HIV stigma. This continuous measure included 10-items assessed on a 4-point Likert scale [47], with a total possible range of 4 10 to 40. Questions addressed whether participants felt bad or unworthy due to their HIV status. The HIV Stigma Scale had an alpha of 0.89 in the DR and 0.90 for Tanzania.

##### Sexual Partner Violence

We utilized an adapted measure of the Conflict Tactics Scale [48] with expanded questions on perpetrator types. We assessed whether participants had experienced any physical and/or sexual violence from intimate (boyfriends, husbands) and sex work partners (new, regular clients) [24] during the last 6 months. Each question had a yes/no response option. A composite score was created and dichotomized into any or no violence.

##### Social Cohesion

We assessed social cohesion using a continuous 11-item measure developed by Kerrigan and colleagues for use among FSW [49, 50]. Participants were asked to rate their agreement or disagreement on a 4-point Likert scale with statements related to mutual aid, support, and trust among sex workers, with a total possible range from 11 to 44. The social cohesion measure had an alpha of 0.84 for the DR and 0.87 for Tanzania.

##### Substance Use

We screened for prior substance use (yes/no) across a range of illicit drugs, including cocaine, crack, heroin, marijuana, club drugs and methamphetamines. We assessed alcohol use frequency and quantity in the last month. We also examined whether drugs and/or alcohol were used during sex work: always, almost always, sometimes, rarely or never. This variable was then categorized into: always/almost always, sometimes and rarely/never.

##### Demographic Variables

We also assessed participants’ demographic characteristics including: age; relationship status; number of children; income per month, including from sex work; number of years in sex work; and average number of clients per week.

### Statistical Analysis

Measurement equivalence is critical for valid group comparisons, such as those done in cross-cultural research. In particular, measures with items that perform differently in different settings can produce scores that do not accurately reflect true differences in the underlying construct or latent trait between these settings. To address this issue, this study uses IRT to estimate item parameters for the DR and Tanzania, empirically test for differentially functioning items and account for any differences during scoring. These steps allow final differential item functioning (DIF)-adjusted scores to accurately reflect unique item characteristics in each setting while aligning scores from each country along a common scale.

#### Factor Analysis

Exploratory factor analysis (EFA) was performed to empirically examine the proposed three domain scale structure generated from formative research in both settings. Factor analysis was also used to determine whether the set of items specified for each domain comprise a unidimensional construct, an important IRT assumption [51]. While no definitive criterion for determining unidimensionality exists, “sufficient” unidimensionality for IRT analysis [52] may be demonstrated if the proportion of the variance explained by the first factor is ≥ 20% [53] and if the ratio of eigenvalues between the first and second factor is ≥ 4 [54]. Strong factor loadings (> 0.40) observed for all items on the first factor also provide further support of sufficient unidimensionality for valid IRT modeling.

#### IRT Analysis

IRT modeling was used to first examine item characteristics (e.g. item discrimination and category location parameters) for each scale domain and to identify potential differences in item performance in the two settings through differential item functioning (DIF) analyses [55, 56]. Statistically significant differences in item parameters were adjusted in a final model to provide DIF-adjusted scores that are comparable across the two settings. IRT modeling and DIF analyses were performed using *IRTPRO Version 4.1*.

#### Graded Response Model (GRM)

GRM was used to estimate one discrimination (*a*) parameter and *k* − 1 boundary location (*b*_1_*... b*_*k-*1_) parameters, where *k* = number of response categories, for each item [57]. The *a* parameter reflects the ability of an item to discriminate among persons with different levels of stigma. Higher *a* values indicate better discrimination. For the ESWS, the GRM created two binary comparisons from the three-category Likert response scale, with first category relative to the last two (*b*_1_) and then the first two categories relative to the last (*b*_2_). GRM analyses revealed generally good discrimination (a > 2.0) and location parameters reflecting broad trait coverage. Four items (all of which were reverse coded) which did not meet these minimum criteria and also showed poor internal consistency in reliability analysis were dropped.

#### Differential Item Functioning (DIF)

DIF was determined using likelihood ratio (LR) difference tests. These tests were used to examine whether item parameters functioned differently by country per each domain. As part of these tests, we first identified a set of “anchor items,” or items that do not demonstrate DIF [58], across DR and Tanzania. Identification of anchor items involved iterative LR tests to identify and exclude items that show DIF. In the initial LR tests for anchor items, an assumption is made that all items other than the item being tested serve as adequate anchors in the initial round. Subsequent LR tests are performed only within the set of preliminary “anchor items” identified by the previous LR test. Additional items identified with DIF are excluded from the anchor set. This process was repeated until the set of anchor items include no items demonstrating DIF. During this process one item was removed from the enacted stigma domain, due to a lack of variation in response patterns in the DR, inhibiting DIF comparisons and analysis.

Using the final set of anchor items, we tested for a difference in discrimination and location parameters by country for each non-anchor item. Statistically significant differences in item parameters based on LR tests determine the items and the parameters that should be modeled separately by country. A final model accounts for the differences by country and provide DIF-adjusted scores taking into account items that demonstrated any DIF.

#### Final Scoring and Distribution

Based on the PROMIS metrics [59], and for ease of interpretation, DIF-adjusted scores were rescaled using a mean of 50 and a standard deviation of 10. When creating the DIF-adjusted scores, the DR sample served as the reference group for all IRT DIF analysis. We generated descriptive statistics for each of these DIF-adjusted ESWS domain scores, including the % maximum and % minimum of each score to examine potential ceiling and floor effects.

#### Reliability and Validity Analyses

Cronbach’s alpha was used to examine internal consistency for each domain and the overall scale [60]. Cronbach’s alpha > 0.70 is used to indicate adequate reliability [60].

Construct validity of the new measure, based on DIF-adjusted scores, was assessed through testing for expected associations with related constructs. Specifically, we conducted bivariate logistic and linear regression analysis examining the association between each ESWS domain. We hypothesized the ESWS domains would be significantly associated with greater levels of depression, anxiety, internalized HIV stigma and sexual partner violence, as well as lower levels of social cohesion.

Characteristics of two cohorts were compared using t-tests and non-parametric Wilcoxon rank-sum tests for continuous variables and chi-squared tests for categorical variables.

## Results

### Characteristics of the Samples

The two cohorts of FSW that participated in the construction of the ESWS scale have both shared and distinct socio-demographic characteristics as seen in Table 1. The median age of participating women was older in the DR (41 years) compared to those in Tanzania (33 years; p < 0.001). A greater percentage of women in the DR were married or cohabitating at the time of the interview (41.23% vs. 29.27%; p < 0.001). The majority of women from both settings had a primary school level of education (56.87% in the DR and 77.07% in Tanzania; p < 0.001). However, more women in the DR had attended secondary school or university (p < 0.001). The median number of children was 2 in Tanzania compared to 3 in the DR (p < 0.001). Similar percentages of women in each setting were the primary income earner in their household (75.36% in the DR and 80.49% in Tanzania). In the DR, sex work represented three-quarters of the monthly income compared to half in Tanzania (p < 0.001). Women in the DR had been sex workers for a significantly longer period (median of 19 years in the DR and 14 years in Tanzania; p < 0.001). In the DR, the median number of clients per week was 3.0 vs. 2.0 in Tanzania (p < 0.001). Substance use was common in both samples, with alcohol use more prevalent in Tanzania (46.83% drank 4 or more times a week vs. 12.80% in the DR) and drug use more prevalent in the DR (26.54% reported prior drug use vs. 4.39% in Tanzania; p < 0.001). Substance use during sex work almost always or always 41.95% in Tanzania vs. 34.60% in the DR (p < 0.001).

**Table 1:** Sociodemographic, occupational and behavioral characteristics of the samples

Variable	Dominican Republic	Tanzania	P-value
	(n = 211)	(n = 205)	
	n (%)	n (%)	
Demographics			
Age in years (median, range)	41 (21–67)	33 (20–55)	< 0.001
Marital status			
Single	105 (49.76)	80 (39.02)	< 0.001
Married or cohabitating	87 (41.23)	60 (29.27)	
Separated, divorced or widowed	19 (9.00)	65 (31.71)	
Education			
No school	9 (4.27)	14 (6.83)	< 0.001
Primary	120 (56.87)	158 (77.07)	
Secondary	71 (33.65)	33 (16.10)	
University	11 (5.21)	0 (0.00)	
Number of children (median, range)	3 (0–9)	2 (0–7)	< 0.001
Occupation			
Monthly income overall (median, range)	USD 146.09 (0–730.46)	USD 43.21 (4.32–38.89)	< 0.001
Proportion of monthly income from sex work (median, range)	0.75 (0–1)	0.50 (0–1)	< 0.001
Main household financial supporter with income:			
No	52 (24.64)	40 (19.51)	0.207
Yes	159 (75.36)	165 (80.49)	
Number of years in sex work (median, range)	19 (1–50)	14 (1–37)	< 0.001
Number of clients per week (median, range)	3 (0–30)	2 (0–21)	< 0.001
Substance use			
Alcohol use			
Never	38 (18.01)	45 (21.95)	< 0.001
Once a month or less	49 (23.22)	10 (4.88)	
2 to 4 times a month	51 (24.17)	10 (4.88)	
2 to 3 times a week	46 (21.80)	44 (21.46)	
4 or more times a week	27 (12.80)	96 (46.83)	
Ever used drugs			
No	155 (73.46)	196 (95.61)	< 0.001
Yes	56 (26.54)	9 (4.39)	
Substance use when meeting clients during the last month			
Never/rarely	73 (34.59)	97 (47.32)	< 0.001
Sometimes	65 (30.81)	22 (10.73)	
Almost always/always	73 (34.60)	86 (41.95)	

### Factor Analysis

Findings from factor analysis guided the development of the final ESWS scale structure, including the development of an additional domain of sex work stigma. Through EFA we observed the presence of two separate factors within the originally conceptualized single internalized sex work stigma domain, which had a total of 12 items. Further review of the data from both the cognitive interview process and EFA indicated that personal feelings related to sex work stigma was manifesting through two separate domains: “shame” or internalized sex work stigma and “dignity” or resisted sex work stigma, each comprised of 6 items. We conducted additional factor analyses and statistical tests to ensure that both domains met the sufficient unidimensionality criteria for valid IRT modeling.

Factor analysis also allowed us to confirm the presence of two additional scale domains, which, as hypothesized included “silence” or anticipated stigma (including 8 items e.g. avoidance of communication about participation in sex work) and “treatment” or enacted stigma (including 12 items e.g. experiences of being mistreated for being a sex worker). Through the EFA process, and in conjunction with reliability analysis, two items were dropped from both the anticipated and enacted sex work stigma domains, respectively, to ensure that all unidimensionality criteria for IRT analysis were met. Factor loadings for items retained after EFA for a given domain are shown in Table 2 were > 0.40.

**Table 2:** Factor analysis per ESWS subdomain across countries

	Dominican Republic (n = 211)				Tanzania (n = 205)			
	Shame (Internalized)	Dignity (Resisted)	Treatment (Enacted)	Silence (Anticipated)	Shame (Internalized)	Dignity (Resisted)	Treatment (Enacted)	Silence (Anticipated)
Number of items	6	6	12	8	6	6	12	8
Proportion of variance explained	80.26%	86.01%	67.42%	82.18%	92.54%	86.05%	73.30%	64.88%
Ratio of Eigen value (1st/2nd factor)	7.32	13.04	6.99	11.40	21.11	15.70	7.42	3.00
Number of items with factor loadings over > 0.40 (range)	6/6 (.62–.89)	6/6 (.78–.92)	12/12 (.54–.89)	8/8 (.61–.96)	6/6 (.83.95)	6/6 (.81–.94)	12/12 (.57–.96)	6/8 (.60–.97)

### Conceptual Model

**Fig. 1:**
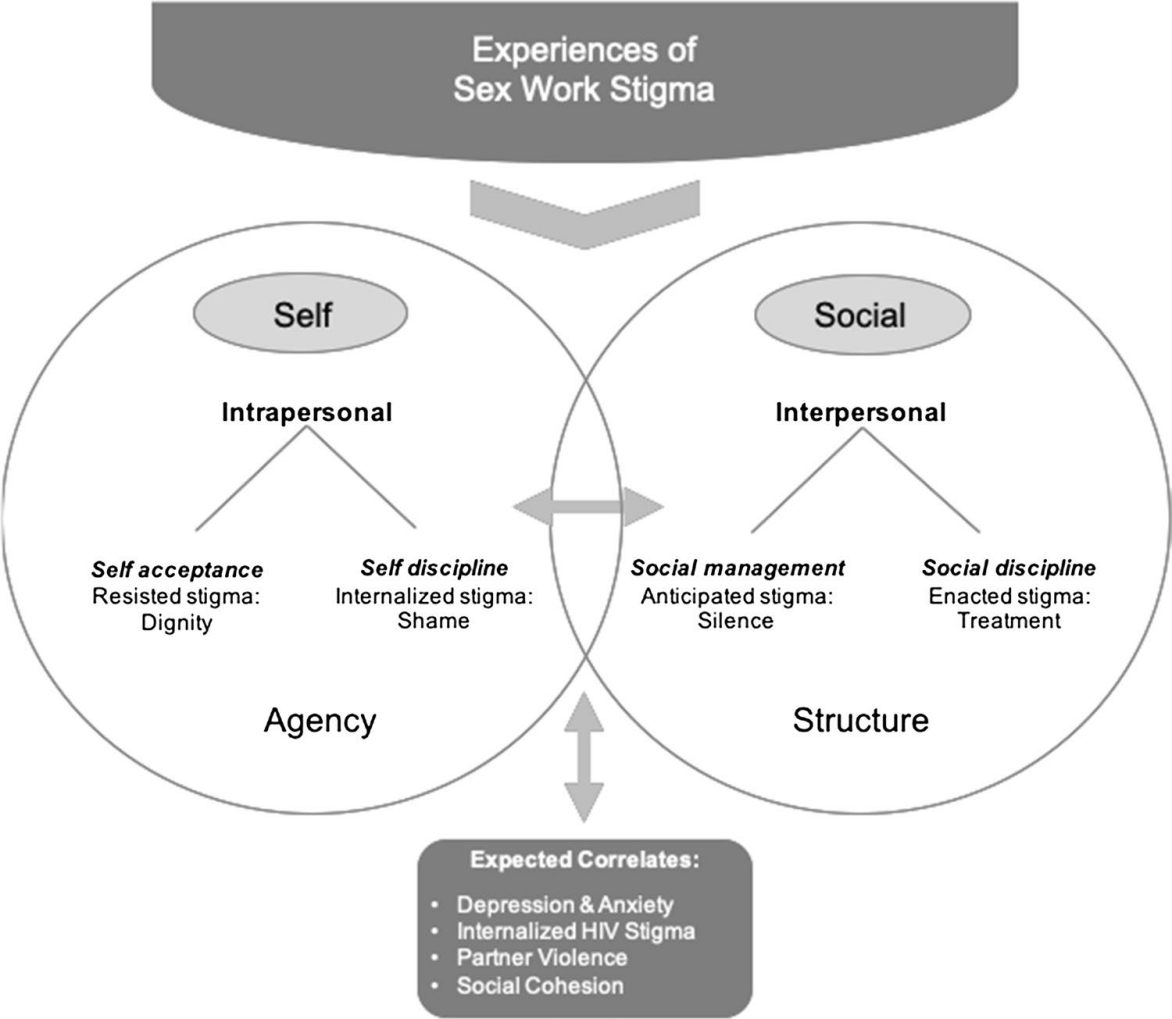
Conceptual model of the experiences of sex work stigma (ESWS) scale

Figure 1 depicts the conceptual model developed for sex work stigma as a result of the in-depth interviews, cognitive debriefing interviews and factor analysis, which led us to the establishment of the final four-domain scale structure. Similar to prior stigma frameworks related to HIV [14], we observed distinct forms and mechanisms of sex work stigma, as seen in, including: internalized, anticipated and enacted stigma. However, we also observed, as noted above, an additional mechanism not often contemplated in existing HIV stigma frameworks, which was the domain of resisted stigma. The mechanisms we observed operate at distinct levels including the intrapersonal (internalized and resisted) and the interpersonal levels (anticipated and enacted). These mechanisms are also situated, as seen in the conceptual model below, in the context of relevant social theoretical constructs discussed earlier, including the dynamics of both self and social discipline, as well as possibilities for challenging stigmatizing identities and inequitable power structures, through individual and collective agency.

### IRT Model Parameters and Differential Item Functioning

IRT modeling of items in each domain indicate good discrimination and coverage of each trait as seen in Table 3. Most items have discrimination (a) parameters ≥ 2. Furthermore, category location parameter values (e.g., b1 and b2) for each item generally differed by values of between 1 and 2, indicating the ability of most items to distinguish differences in a broad region of the underlying domain trait (i.e., between 1 and 2 standard deviation of the DR sample distribution). For example, b1 = 0.39 and b2 = 2.25 for the “rejected” item in the shame domain suggests that this item provides optimal measurement of this domain in the region between 0.39 and 2.25 standard deviation above the DR group mean of 0. Differential item functioning analysis found that most items in each domain did not show DIF. Most DIF items were significant for location parameter, although three items also showed DIF in discrimination. During this process, we identified one original item (“Have not greeted you”) in the treatment (enacted) stigma domain with limited variability, constraining the DIF analysis, which was subsequently dropped.

**Table 3:** Graded model item parameter estimates from IRT analysis across countries

Domains and Items	Dominican Republic and Tanzania^+^			DIF
	a (discrimination)	b_1_ (location)	b_2_ (location)	
Shame (internalized)				
Rejected	2.39	0.39	2.25	
Excluded	2.61	0.43	2.28	
Different	2.53	0.24	2.10	
Ashamed				
Dominican Republic	1.21	0.03	1.53	a & b
Tanzania	1.93	− 0.30	1.66	
Humiliated				
Dominican Republic	3.81	0.40	1.81	b
Tanzania		− 0.39	1.75	
Frustrated				
Dominican Republic	2.33	0.47	1.97	b
Tanzania		0.10	2.75	
Dignity (resisted)				
Valued	2.54	− 1.27	− 0.04	
Comfortable	2.95	− 1.04	0.11	
Accepted	2.75	− 1.30	0.03	
At peace	2.33	− 1.43	− 0.19	
Happy	3.32	− 1.13	0.19	
Proud				
Dominican Republic	3.85	− 0.53	− 0.04	b
Tanzania		− 0.71	0.34	
Treatment (enacted)				
Distanced themselves from you	2.61	0.76	2.47	
Excluded you from groups	2.00	1.45	2.91	
Laughed at you	4.95	0.54	1.88	
Called you names	2.81	0.06	1.88	
Ignored you	3.92	0.25	2.21	
Humiliated you	3.97	0.55	1.92	
Mistreated you	2.76	0.64	2.62	
Criticized you				
Dominican Republic	2.52	− 0.05	1.76	b
Tanzania		0.64	2.22	
Treated you differently from other women				
Dominican Republic	2.25	0.31	2.39	b
Tanzania		1.16	3.04	
Silence (anticipated)				
You have tried to make sure that no one knows that you do sex work	2.99	− 1.00	− 0.17	
You have done everything you can to keep sex work a secret	6.24	− 0.96	− 0.38	
You have ensured that no one in your community finds out about your sex work	2.68	− 1.00	− 0.31	
You have avoided talking about sex work	2.62	− 1.02	0.00	
You have hidden from your family that you do sex work				
Dominican Republic	2.59	− 0.78	0.14	a & b
Tanzania	1.29	− 3.63	− 2.06	
You have denied that you have worked as a sex worker				
Dominican Republic	2.84	− 0.44	0.46	a & b
Tanzania	0.54	0.00	2.62	

^+^Discrimination (a) and location (b_1_ and b_2_) parameters are the same for each country unless otherwise noted in the table

Ultimately, DIF analysis indicated most parameters were consistent in measuring the latent trait of sex work stigma across the DR and Tanzania samples. Nevertheless, the results also highlighted the items for which certain parameters of discrimination and/or location varied across the samples and thus the need to calibrate those items in order to develop a final DIF-adjusted score in each domain that accounts for these parameter differences and is therefore comparable across the samples.

### Reliability and Distribution

Table 4 presents the final ESWS scale which includes a total of 27 items across the 4 scale domains: shame (internalized, 6 items), dignity (resisted, 6 items), silence (anticipated, 6 items) and treatment (enacted, 9 items) sex work stigma. Reliability for all final ESWS domains was > 0.80, with alphas ranging from 0.81 for shame (internalized sex work stigma) in the DR to 0.93 for treatment (enacted sex work stigma) in Tanzania.

**Table 4:** Final items reliability, ceiling and floor effects, and distribution of ESWS domains using final DIF-adjusted IRT scores

	Dominican Republic (n = 211)					Tanzania (n = 205)			
	Shame (Internalized)	Dignity (Resisted)	Treatment (Enacted)	Silence (Anticipated)		Shame (Internalized)	Dignity (Resisted)	Treatment (Enacted)	Silence (Anticipated)
Number of items	6	6	9	6		6	6	9	6
Cronbach’s Alpha	0.81	0.89	0.90	0.90		0.91	0.89	0.93	0.88
Floor Effect^a^	27.49	2.84	32.23	9.48		17.07	31.22	35.61	2.93
Ceiling Effect^b^	0.95	25.59	0.00	18.01		3.90	3.41	0.49	24.39
Mean (SD)	50.00 (8.85)	50.01 (9.20)	50.00 (9.13)	49.98 (9.12)		53.33 (11.82)	35.11 (9.15)	51.33 (10.78)	56.14 (16.39)
Median (range)	49.04 (39.61–77.61)	48.73 (29.48–62.16)	50.71 (39.30–73.76)	51.21 (31.66–62.26)		53.79 (36.05–82.34)	34.62 (24.79–59.27)	51.18 (39.39–84.52)	64.68 (18.09–73.56)

^a^Percentage of participants scoring the lowest possible scale score

^b^Percentage of participants scoring the highest possible scale score

The distribution of the DIF-adjusted rescaled score for each domain in the DR and Tanzania is also found below, whereas higher scores on the shame, silence and treatment domains represent greater levels of stigma in relation to sex work and higher scores on the dignity domain indicate greater levels of resistance to sex work stigma. Table 4 also shows the percentage of women that had a score at the minimum and maximum score for a given domain, reflecting floor and ceiling effects. We observed moderate ceiling effects for silence (anticipated stigma) domain and floor effects for the shame (internalized) and treatment (enacted) stigma domains in both countries. While most patterns of floor and ceiling effects were similar across countries, we observed a distinct pattern for dignity (resisted stigma), with a significantly greater percentage of women scoring at the maximum in the DR (25.59%) compared to Tanzania (3.41%).

### Validity

Table 5 presents results from the construct validity analyses of the ESWS domains based on expected associations with depression, anxiety, HIV stigma, sexual partner violence and social cohesion. We found that the scale domains were associated with a greater number of these outcomes in the DR compared to Tanzania. However, all domains were associated with at least one of the expected outcomes across the two countries. The internalized (shame) sex work stigma domain was significantly associated with all of the outcomes examined in the DR and Tanzania in the expected direction (e.g. greater depression, anxiety, HIV stigma and partner violence and less social cohesion). Dignity of resisted sex work stigma was significantly associated with all outcomes examined in the DR, but only marginally associated with greater cohesion in Tanzania. Treatment or enacted sex work stigma was significantly associated with all examined outcomes except social cohesion in the DR and significantly associated with sexual partner violence in Tanzania. Higher scores on the silence (anticipated sex work stigma) domain were significantly associated with lower levels of social cohesion in both the DR and Tanzania, and greater levels of shame or internalized HIV stigma in the DR only.

**Table 5:** Bivariate analysis assessing the external validity of the ESWS scale domains

Outcomes	Dominican Republic (n = 211)				Tanzania (n = 205)			
	Shame (Internalized)	Dignity (Resisted)	Treatment (Enacted)	Silence (Anticipated)	Shame (Internalized)	Dignity (Resisted)	Treatment (Enacted)	Silence (Anticipated)
	OR (SE)	OR (SE)	OR (SE)	OR (SE)	OR (SE)	OR (SE)	OR (SE)	OR (SE)
Depression^a^	1.08*** (0.02)	0.95*** (0.02)	1.08*** (0.02)	0.99 (0.02)	1.06** (0.03)	0.99 (0.04)	1.07* (0.04)	1.02 (0.03)
Anxiety^b^	1.08*** (0.02)	0.95*** (0.02)	1.10*** (0.02)	0.97* (0.02)	1.05** (0.02)	1.01 (0.03)	1.02 (0.02)	1.01 (0.02)
Violence^c^	1.10*** (0.03)	0.92*** (0.02)	1.14*** (0.03)	0.97 (0.02)	1.04*** (0.01)	1.01 (0.02)	1.06*** (0.02)	1.00 (0.01)

	β (SE)	β (SE)	β (SE)	β (SE)	β (SE)	β (SE)	β (SE)	β (SE)
HIV Stigma	0.12*** (0.02)	− 0.11*** (0.02)	0.09*** (0.02)	0.07*** (0.02)	0.12*** (0.04)	− 0.06 (0.05)	0.07 (0.05)	0.04 (0.03)
Social Cohesion	− 0.10*** (0.03)	0.12*** (0.03)	− 0.05 (0.03)	− 0.12*** (0.03)	− 0.07** (0.03)	0.07* (0.04)	− 0.005 (0.03)	− 0.10*** (0.02)

***p < 0.01, **p < 0.05, *p < 0.10

^a^Moderate to severe depression (PHQ-9)

^b^Borderline abnormal/abnormal anxiety (HADS-A)

^c^Any sexual or physical violence from new/regular client or intimate partner

## Discussion

This work represents the first rigorous scale development process related to sex work stigma across contexts. Guided by social theory and the perspectives of sex workers themselves through extensive formative research [61] and grounded in long-standing community partnerships [62], the 4-domain ESWS scale was found to be valid and reliable across two distinct geographic and sociocultural settings. These four domains represent the multiple dimensions of the experience of sex work stigma. While they share commonalities with prior HIV stigma measurement work [14] (e.g. internalized, anticipated and enacted sex work stigma), they also reflect unique aspects and contributions to the field with the emergence of a resisted stigma domain. Each of these domains was also significantly associated with at least one related construct per setting, including: depression, anxiety, HIV stigma, sexual partner violence and/or social cohesion. As each of these constructs has also been previously found to be associated with HIV-related outcomes, the ESWS scale will be an important measurement tool in future assessments of the social determinants of HIV, and in the development and evaluations of interventions seeking to address these underlying determinants. Specific next steps for our ongoing longitudinal study among FSW in the DR and Tanzania will include an assessment of the ESWS scale’s predictive validity in terms of its relationship with HIV outcomes (e.g. retention in care, ART adherence and viral suppression). These analyses will also seek to illuminate the mechanisms and pathways between sex work stigma and HIV outcomes, as well as elucidate how sex work stigma may intersect with other social stigmas (e.g. HIV stigma) to impact HIV and other health outcomes.

The ESWS scale makes important theoretical contributions to the literature regarding stigma as a social determinant of health [63]. The measure highlights the importance of both self (shame) and social stigmatization (silence, treatment), as well as the possibilities for transformation of subjectivities (resistance) [64] as reflected in the work of critical social theorists. While recent growing attention to structural stigma is warranted [65], attention to the interplay between structure and agency [34] including in relation to stigma [33], is also critical to a nuanced understanding of how these dynamics operate in people’s everyday lives. Emphasis on the interplay between the self and social practices also opens up new spaces for intervention, including the possibilities of resistance of and challenges to stigmatized identities at both an individual and collective level. As the ESWS scale is used in subsequent research on the social determinants of HIV, it will allow for a greater understanding of when and how, as well as under what social and structural conditions, such resistance emerges and is sustained, and its influence on the health and well-being of sex workers across contexts. For example, here we observed greater levels of dignity (resisted sex work stigma) in the DR, where there is a long history of community-driven HIV prevention interventions among FSW as compared to the more recent emergence of community empowerment-based approaches to HIV prevention and care among FSW in Iringa, Tanzania [62].

This study also represents a significant methodological advance through the development of a cross-culturally valid measure. Formative qualitative research performed in both the DR and Tanzania allowed for the development of a conceptual framework for sex work stigma that is applicable across settings. In addition, our study included extensive IRT analyses to examine item discrimination and location parameters, including the evaluation of DIF across settings. In particular, the identification and modeling of items that demonstrated statistically significant DIF separately for each country allows for the accounting of these differences in the final scores [66] and the appropriate use of these DIF-adjusted scores in analyses across distinct geographic and cultural contexts. Most items evaluated did not show DIF, which further supports the cross-cultural applicability of the performance of the final measure.

This work is not without its limitations. The cohort participants involved in scale development included cisgender FSW only. In turn, it is not possible to generalize findings to transgender female, transgender male or cisgender male sex workers. Additionally, all study participants were HIV-positive. The measure development process focused on participants’ experiences as sex workers. However, interviews did reveal the double burden and intersectional experience of being a sex worker and living with HIV [61]. While we believe the measure is relevant for HIV-negative sex workers, validation in this population will also be important. Additionally, the scale focused on individual-level self-report of sex work stigma experiences rather than accounting for stigma at a socio-structural or institutional level. Lastly, while the measure was reliable and valid in both settings, it showed stronger validity in the DR compared to Tanzania. Validation of the ESWS scale in other geographic settings will help to further establish and/or adapt and refine the measure for cross-context analysis. In addition, while this study illustrated a method for examining and addressing measurement differences between DR and Tanzania, measurement equivalence of the ESWS in other geographic settings will need to be empirically tested in future studies.

As interest in examining the influence of stigmas which intersect with HIV stigma has grown, the need for valid and reliable measures of these stigmas has also increased. The ESWS scale represents a critical tool for documenting multi-dimensional aspects of occupational stigma experienced by sex workers which can be used across contexts.

## Data Availability

Data is available upon request from the first author.

## Code Availability

Coded data is available upon request from the first author.
