# Correction to: Development of the Experiences of Sex Work Stigma Scale Using Item Response Theory: Implications for Research on the Social Determinants of HIV

**DOI:** 10.1007/s10461-021-03298-6

**Published:** 2021-05-21

**Authors:** Deanna Kerrigan, Tahilin S. Karver, Clare Barrington, Wendy Davis, Yeycy Donastorg, Martha Perez, Hoisex Gomez, Jessie Mbwambo, Samuel Likindikoki, Catherine Shembilu, Andrea Mantsios, S. Wilson Beckham, Noya Galai, Kitty S. Chan

**Affiliations:** 1 Department of Prevention and Community Health, Milken Institute School of Public Health, George Washington University, 950 New Hampshire Avenue NW, Washington, DC, 20052 USA; 2 Department of Health Behavior, University of North Carolina, Chapel Hill, USA; 3 Department of Health, Behavior and Society, Johns Hopkins University, Baltimore, USA; 4 Department of Epidemiology, Johns Hopkins University, Baltimore, USA; 5 Instituto Dermatologico Dominicano y Cirugia de Piel (IDCP), Santo Domingo, Dominican Republic; 6 Muhimbili University of Health and Allied Sciences (MUHAS), Dar es Salaam, Tanzania; 7 Public Health Innovation & Action, New York, NY USA; 8 University of Haifa, Haifa, Israel; 9 Medstar Health Research Institute, Washington DC, USA

The article “Development of the Experiences of Sex Work Stigma Scale Using Item Response Theory: Implications for Research on the Social Determinants of HIV”, written by Deanna Kerrigan, Tahilin S. Karver, Clare Barrington, Wendy Davis, Yeycy Donastorg, Martha Perez, Hoisex Gomez, Jessie Mbwambo, Samuel Likindikoki, Catherine Shembilu, Andrea Mantsios, S. Wilson Beckham, Noya Galai, Kitty S. Chan was originally published electronically on the publisher’s internet portal on 17th March 2021 without open access. With the author(s)’ decision to opt for Open Choice the copyright of the article changed on 29th April 2021 to © The Author(s) 2021 and the article is forthwith distributed under a Creative Commons Attribution.

“The original article has been corrected.”

